# Possible Contribution of Oral Microbiota in the Osteonecrosis of the Jaw Induced by Zoledronate or Denosumab: A Preliminary Study

**DOI:** 10.3390/biomedicines14040786

**Published:** 2026-03-30

**Authors:** Francesco Maria Erovigni, Alessandra Manca, Virginia Moscone, Miriam Antonucci, Valeria Ghisetti, Giorgia Menegatti, Francesco Chiara, Jacopo Mula, Alice Palermiti, Vittorio Fusco, Lorenzo Bianchi, Paolo Arduino, Antonio D’Avolio, Jessica Cusato

**Affiliations:** 1 Presidio Dental School, AOU Città Della Salute e Della Scienza di Torino, 10126 Turin, Italy; francesco.erovigni@unito.it (F.M.E.); virginia.moscone96@gmail.com (V.M.); drlorenzobianchi@gmail.com (L.B.); paolo.arduino@unito.it (P.A.); 2 Laboratory of Clinical Pharmacology and Pharmacogenetics, Department of Medical Sciences, University of Turin, 10149 Turin, Italy; giorgia.menegatti@unito.it (G.M.); jacopo.mula@unito.it (J.M.); alice.palermiti@unito.it (A.P.); antonio.davolio@unito.it (A.D.); jessica.cusato@unito.it (J.C.); 3 Azienda Sanitaria Locale (ASL) Città di Torino, Amedeo di Savoia Hospital, 10149 Turin, Italy; miriam.antonucci@aslcittaditorino.it (M.A.); valeria.ghisetti@aslcittaditorino.it (V.G.); 4 Laboratory of Clinical Pharmacology San Luigi A.O.U., Department of Clinical and Biological Sciences, University of Turin, 10043 Orbassano, Italy; francesco.chiara@unito.it; 5 Oncology Unit, Department of Medicine and Translational Medicine Unit, DAIRI—Department of Integration, Research and Innovation “SS Antonio e Biagio e C.Arrigo” Hospital, Azienda Ospedaliero-Universitaria “SS Antonio e Biagio e C.Arrigo”, 15121 Alessandria, Italy

**Keywords:** MRONJ, antibiotics, infections, saliva microbioma, antiresorptive-drug-adverse reaction

## Abstract

**Background/Objectives**: Medication-related osteonecrosis of the jaw (MRONJ) is a clinically significant side effect related to antiresorptive therapies, such as denosumab and bisphosphonates. MRONJ may develop following oral surgical procedures or spontaneously. Although the pathophysiological processes underlying MRONJ are not well clarified, infections, commonly occurring after oral surgery, seem to have an important contribution in its development. Consequently, the role of the oral microbiota warrants investigation. This study investigates the possible contribution of the salivary microbiota to the onset of osteonecrosis in subjects treated with zoledronate or denosumab. **Methods**: Three groups of subjects were analyzed: patients treated with zoledronate or denosumab who had developed MRONJ (cases); those who did not (controls) and healthy subjects. Oral microbioma was evaluated through next-generation sequencing. **Results**: A total of 55 individuals were enrolled: 16 healthy subjects (29.1%), 21 controls (38.2%), and 18 cases (32.7%). Differences in the abundance of certain bacterial taxa were observed both among the three groups and in pairwise comparisons. Furthermore, a cut-off value of 5.51% for *Streptococcus* spp. was identified as being associated with the development of MRONJ. **Conclusions**: For the first time, this preliminary study highlights differences in the salivary microbiota among healthy subjects, controls, and cases, suggesting a potential cut-off value for *Streptococcus* spp. Despite the limited sample size, these findings provide initial insights. Further studies in larger cohorts are warranted.

## 1. Introduction

Osteonecrosis of the jaw (ONJ) is severe pathology which can affect both jaws, albeit more frequently in the mandible [1].

Over time, both the terminology and the understanding of ONJ have evolved. The term “BRONJ” (bisphosphonate-related osteonecrosis of the jaw) was initially used to describe cases associated with bisphosphonate therapy. However, with the introduction of additional drugs implicated in its pathogenesis, the broader term “medication-related osteonecrosis of the jaw” (MRONJ) was adopted to encompass a wider range of therapeutic agents, including bisphosphonates, denosumab, and other antiresorptive or antiangiogenic agents [1].

MRONJ is described as one or more necrotic bone lesions, typically exposed in the buccal cavity for at least eight weeks, in subjects receiving antiresorptive or antiangiogenic therapies for conditions such as Paget’s disease, osteoporosis or primary and metastatic bone tissue malignancies [1,2,3,4,5,6,7].

Over time, the nosological definition of MRONJ has been widely debated. The diagnostic criteria proposed by the American Association of Oral and Maxillofacial Surgeons (AAOMS) have helped standardize the definition; however, several limitations have emerged [8,9]. First, exposed necrotic bone or intra-/extraoral fistulas, often late manifestations, are not always present in the initial stages of the pathology, potentially delaying diagnosis in a subset of patients. Second, the requirement for these signs to persist for at least eight weeks may further delay treatment initiation, thereby limiting therapeutic effectiveness. Third, the recognition of a “stage 0,” characterized by nonspecific clinical or radiological findings without exposed bone, introduces a discrepancy within the AAOMS classification and poses diagnostic challenges. Finally, the evolving understanding of MRONJ pathogenesis, including the role of additional drugs and local or systemic risk factors, supports the need for a more inclusive and flexible definition. Disease staging is focused on the gravity of clinical symptoms and the extent of radiological findings [4]. The AAOMS staging system is currently the most widely used. Stage 0 is described as the lack of clinically evident necrotic bone, with only specific symptoms or clinical and/or radiographic findings. Stage I includes exposed and necrotic bone, or fistulae probing to bone, in asymptomatic patients without presence of soft-tissue inflammation or infection. Stage II is defined by exposed infected necrotic bone, characterized by pain and inflammation of the surrounding or regional soft tissues, with or without purulent discharge. Finally, stage III consists of exposed and necrotic bone, or fistulae probing to bone, associated with pain and infection, as well as at least one among pathological fracture, extraoral fistula, oroantral fistula, or radiographic evidence of osteolysis in the inferior border of the mandible or maxillary sinus [10].

Patients may present with pain, inflammation, erythema, and suppuration. Although ONJ can occur spontaneously, it is frequently associated with invasive dental procedures, such as tooth extraction or implant placement, particularly in patients treated with bisphosphonates, antiresorptive biologic agents, or other related medications [3].

According to the “inside–outside” theory, both denosumab and bisphosphonates reduce bone turnover by suppressing osteoclastic activity [11]. In the presence of jaw microdamage caused by local inflammation, impaired bone remodeling may lead to bone tissue necrosis and prolonged exposure of bone to pathogenic microorganisms [12]. According to previous studies, bone necrosis is associated with inflammatory processes and may precede clinical onset [13]. These results highlight bone exposure should not be considered a prerequisite for the development of bone necrosis. Furthermore, it has been proposed that the bone resorption inhibition in mechanically stressed jawbones is mediated by distinct antiresorptive agents, such as zoledronate and denosumab, which differ in their pharmacodynamic profiles. An alternative hypothesis, known as the “outside–inside” theory, postulates that local immune suppression induced by antiresorptive therapies, in combination with mucosal or dental lesions, may facilitate the development of local infection or inflammation involving the underlying bone, ultimately leading to osteonecrosis. Dental conditions appear to represent important risk factors for MRONJ; accordingly, maintaining good oral hygiene is strongly recommended in cancer patients to reduce the risk of disease onset [14]. Furthermore, previous studies have shown that complex polymicrobial biofilms colonize exposed bone surfaces in MRONJ, potentially contributing to treatment failure [15]. Bisphosphonate treatment may alter the oral environment and promote increased bacterial adhesion to bone surfaces exposed to or coated with these agents, potentially contributing to the development of BRONJ. Bone exposure during surgical procedures, such as tooth extraction, may act as a triggering event, facilitating bacterial invasion. Consequently, this creates a microenvironment that favors infection at the bone surface, supporting a potential role of microbial factors in MRONJ pathogenesis [16].

Among skeletal sites, the jaws are particularly susceptible to infection. This is partly due to the relatively thin mucoperiosteal covering of the maxillary and mandibular bones, which provides less protection compared to the thicker soft tissue and skin layers surrounding other bones. In addition, masticatory function and dental activity expose the jaws to repeated microtrauma. In this context, the replacement of alveolar bone is up to ten times higher than that of long bones, potentially leading to greater local accumulation of bisphosphonates compared with other skeletal sites [11].

In contrast to other body sites, the buccal cavity harbors a heterogeneous microbial ecosystem rich in bacteria and fungi, which can readily colonize exposed bone surfaces, increasing the risk of biofilm-mediated disease. Furthermore, immunosuppressive therapies are frequently administered to cancer patients, rendering them more susceptible to infections. The affected bone may act as a reservoir for periapical and periodontal pathogens, thereby sustaining chronic inflammatory and immune responses [16].

In this context, it is important to highlight that basically these patients are treated with antibiotics that could select specific bacteria on teeth [17,18].

Currently, limited data are available on specific bacterial taxa that may serve as potential predictive risk factors for the onset of MRONJ. Therefore, the aim of this study was to investigate the role of the salivary microbiota in the development of osteonecrosis in patients treated with zoledronate or denosumab, by comparing their oral microbial profiles with those of healthy subjects.

## 2. Materials and Methods

### 2.1. Study Design and Patient Characteristics

This is part of a prospective study performed at the S.C. Riabilitazione Orale Protesi Max. Facc. E Implant. Dentaria–U Servizio di Chirurgia Orale, Torino, Italy, Città della Salute e della Scienza, was performed. Osteoporotic or oncologic patients treated with zoledronate (high dose: 4 mg/4 weeks; dose, low dose: 5 mg/1 year) or denosumab (high dose: 120 mg/4 weeks; low dose: 60 mg/6 months) collected from December 2022 to March 2024 were considered eligible for the present study. Baseline collected data are age, gender, BMI, hemato-chemical analyses if available, and information about other medications and co-morbidities. All patients started antibiotic therapy 2 days before surgery. After the exclusion of patients with other co-morbidities, they were divided into two groups: those who had developed MRONJ (cases) and those who did not (controls). Inclusion criteria were individuals aged >18 years, treated with denosumab or zoledronate, and subject to surgery: controls for tooth extraction, cases for sequestrectomy.

Local Ethic Committee approved the study (number of protocol 427/2022) and it was conducted in agreement with the Helsinki Declaration. All subjects involved in the study provided written informed consent for the enrolment.

### 2.2. Microbioma: Next Generation Sequencing

#### 2.2.1. Sampling and Nucleic Acid Isolation

Oral bacterial DNA was collected from saliva samples using the LolliSponge™ saliva self-collection device (Copan, Brescia, Italy). The collected samples were immediately centrifuged at 1500 rpm and stored at −80 °C until nucleic acid extraction. Prior to extraction, each sample was treated with Lysis/Binding Buffer (Roche, Monza, Italy) as part of the pre-analytical protocol. Bacterial DNA was subsequently extracted using the MagNA Pure 24 system (Roche, Monza, Italy) with a dedicated microbiology panel (https://diagnostics.roche.com/content/dam/diagnostics/Blueprint/en/pdf/rmd/MagNA-Pure-24-Protocols.pdf, accessed on 24 March 2026).

#### 2.2.2. Quantitative Polymerase Chain Reaction for 16S rRNA Next-Generation Sequencing

The next step involved amplification of specific bacterial DNA regions (V1–V2, V3–V4, V5–V6, and V7–V9 regions of the 16S rRNA gene) by PCR using the SimpliAmp system (Thermo Fisher Scientific, Waltham, MA, USA) and a Microbiome Panel Kit (4bases, Chieti, Italy). Subsequently, all samples were barcoded using Barcode Set 1–16 (4bases, Chieti, Italy).

Amplicon libraries were then purified using Agencourt AMPure XP beads (Beckman Coulter, Milan, Italy), adding 40 μL to each LowBind tube according to the manufacturer’s instructions. DNA concentration was quantified fluorometrically using the Qubit™ 4 Fluorometer (Invitrogen, Thermo Fisher Scientific, Waltham, MA, USA) with the Qubit dsDNA HS Assay Kit.

Libraries were subsequently amplified and pooled for template preparation, reaching a final concentration of 100 pM. Amplicon libraries were sequenced on a 520 chip using the Ion Torrent S5 system (Thermo Fisher Scientific, Waltham, MA, USA), following the manufacturer’s instructions.

After sequencing, quality-filtered data were exported as BAM files and analyzed using One Codex software (One Codex, Wilmington, DE, USA, https://www.onecodex.com/, accessed on 24 March 2026).

One Codex performs a two-step abundance-based filtering designed to minimize false positives. Any organism with read counts <0.00005× the total is reassigned to its parent taxon. Any genus-level or lower taxon with <0.01× the abundance of its parent is also reassigned. Filtering based on relative abundance thresholds, rather than fixed Phred score trimming or chimera removal (which are not part of the One Codex Targeted Loci pipeline). Taxonomic classification considers alignment-based (not k-mer-based) for Targeted Loci analysis; it is performed read-by-read and matched to curated marker-gene sequences in the Targeted Loci Database (~250,000 records spanning bacteria, archaea, fungi, protists, algae). One Codex calculates taxonomic relative abundances directly by normalizing the number of reads assigned to each taxon to the total number of classified reads. This normalization step is intrinsic to the One Codex Targeted Loci workflow and is applied automatically after high-sensitivity alignment and taxonomic assignment. No additional external normalization (e.g., rarefaction or scaling methods) is required, as the platform outputs already normalized proportional abundances suitable for downstream interpretation. Normalization is therefore direct and intrinsic to One Codex output. One Codex does not use OTU clustering (no 97% similarity grouping). It also does not use ASV-denoising algorithms (e.g., DADA2, Deblur).

Instead, it performs deterministic per-read alignment to curated reference marker genes.

This produces a taxonomic resolution equivalent to an ASV-level approach, but without clustering or statistical denoising.

### 2.3. Statistical Analysis

Statistical analyses were performed using IBM SPSS Statistics version 28.0 for Windows (IBM Corp., Chicago, IL, USA). Continuous variables are presented as median and interquartile range (IQR; 25th–75th percentile), while categorical variables are expressed as frequencies and percentages.

The normality of continuous variables was assessed using the Shapiro–Wilk test. Comparisons between groups were performed using the Mann–Whitney U test or the Kruskal–Wallis test, as appropriate. Correlations between continuous variables were evaluated using Spearman’s rank correlation coefficient. Associations between categorical variables were assessed using the chi-square (χ^2^) test.

Receiver operating characteristic (ROC) curve analysis was performed to find microbiota-associated cut-off values predictive of MRONJ development.

## 3. Results

### 3.1. Characteristics of the Enrolled Patients

A total of 55 subjects were enrolled: 18 patients (32.7%) with osteonecrosis of the jaw receiving antiresorptive therapy (cases), 21 patients (38.2%) receiving antiresorptive therapy without osteonecrosis (controls), and 16 healthy individuals (29.1%). Their baseline characteristics are summarized in Table 1.

Patients were treated with antiresorptive drug therapy for a median time of 24 (IQR 9; 53.5) months and time since last drug administration was 5 (IQR 2; 10) months.

### 3.2. Microbioma Analysis

The presence of different bacterial taxa was evaluated by comparing both the three groups (healthy subjects, controls, and cases) and the two groups (controls and cases). No significant differences were observed between controls and cases, whereas differences were identified when comparing all three groups, as reported in Table 2.

**Figure 1:**
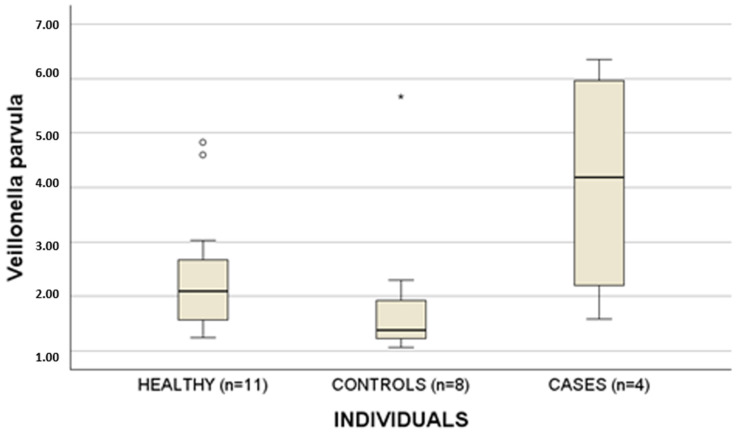
Differences in terms of percentage of *Veillonella parvula* among the three groups (healthy, controls and cases). Circles (○) represent outliers, and asterisks (*) represent extreme outliers.

**Figure 2:**
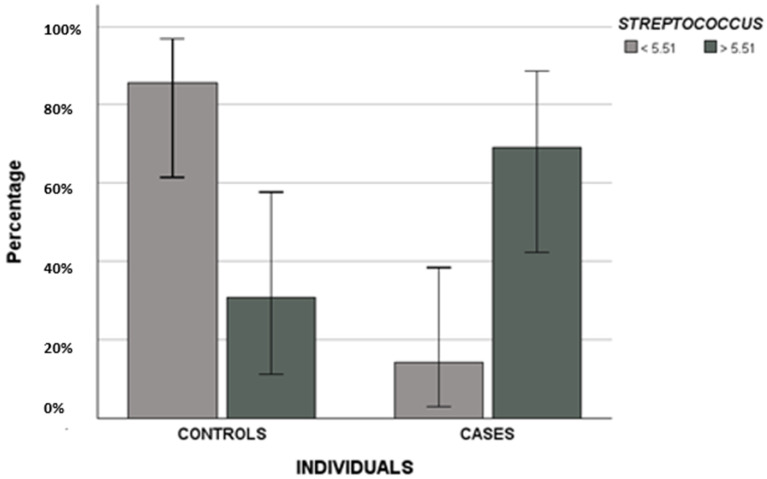
Differences in terms of the *cut-off* value of 5.51% of *Streptococcus* spp. between the two groups (controls and cases).

**Figure 3:**
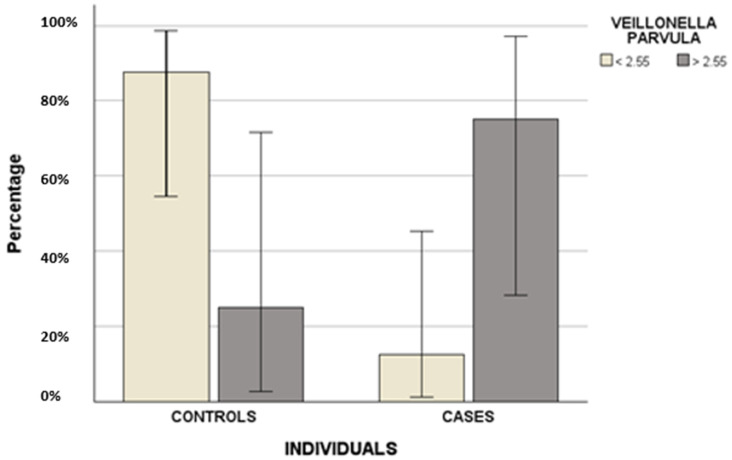
Differences in terms of the *cut-off* value of 2.55% of *Veillonella parvula* between the two groups (controls and cases).

Then, the percentage of bacteria was evaluated as a linear variable: differences were highlighted comparing the three groups (Table 3, Figure 1) and two groups (Table 4, Figure 2 and Figure 3). +, ++ and +++ indicate the abundance of bacteria.

Furthermore, Table 5 reported the ROC analysis performed to define microbioma-associated cut-off values predicting MRONJ development.

## 4. Discussion

Some studies have highlighted the importance of the microbiota in MRONJ. For example, Kim et al. analyzed the oral microbiota of 12 patients, suggesting differences in microbial composition between control subjects and patient groups [19]. Another study reported a predominance of *Firmicutes* in individuals who developed MRONJ compared to controls, along with the expression of several inflammation-related cytokines [20].

Here, for the first time, albeit in a small cohort, we demonstrated differences in oral microbiota among healthy subjects, controls (patients treated with bisphosphonates or denosumab who did not develop MRONJ), and cases (patients treated with bisphosphonates or denosumab who developed MRONJ). In particular, we observed differences among the three groups in terms of bacterial presence (yes/no; dichotomous variable), as shown in Table 2, as well as in relative abundance (%), as reported in Table 3. Furthermore, differences between cases and controls were suggested for *Veillonella parvula*, *Streptococcus* spp., and the *Terrabacteria* group. Obviously, these are preliminary data which have to be confirmed in larger cohorts of patients.

The study by Kim et al. showed results similar to ours regarding *Veillonella* (10). In particular, we found *Veillonella parvula* to be more abundant in cases than in controls. This bacterium is a Gram-negative commensal microorganism present in dental caries and periodontitis. It can act as an opportunistic pathogen by colonizing dental plaque, promoting multispecies growth, and playing an important role in lactic acid fermentation [21].

Furthermore, they suggested that *Streptococcus* spp. were predominant in all samples; however, we observed a higher abundance in cases compared to controls, consistent with the findings of Jelin-Uhlig [22].

The authors highlighted that saccharolytic bacteria such as streptococci (Gram-positive, non-motile, non-spore-forming, and catalase-negative) are commonly present in periodontal disease and MRONJ, suggesting that they may produce an acidic setting that can hinder wound healing [16,23]. Moreover, *Streptococcus* spp. are known to establish intra- and intergeneric co-clustering with other bacteria, such as *Veillonella* spp., contributing to the formation of initial colonies [20]. Thus, the authors conclude that the oral cavity harbors several microorganisms associated with periodontitis, and that their complicate interplay, co-aggregation, and opportunistic behavior may affect the occurrence and progression of MRONJ [16,24,25,26,27].

Another important aspect of our work is the preliminary ROC analysis on *Veillonella parvula*, *Streptococcus* and *Terrabacteria* group in order to define, for the first time, specific cut-off values predicting MRONJ development: we suggested 5.51% for *Streptococcus*, 2.55% for *Veillonella parvula* and 4.1% for the *Terrabacteria group*. In particular, the only predictive factor remained in the final regression model predicting MRONJ occurrence was the cut-off value of 5. 51% for *Streptococcus* spp.

This is an exploratory study; therefore, the statistical conclusions should be interpreted with caution. We hypothesize that the interaction between microbial communities and antiresorptive therapies (e.g., bisphosphonates and denosumab) may alter bone turnover and local immune responses, thereby creating a microenvironment more susceptible to infection and impaired healing. Several factors, including antibiotic therapy, periodontal status, oral hygiene, diet, systemic conditions, and smoking habits, may significantly influence oral microbiome composition and act as potential confounders. In our study, all patients started antibiotic therapy two days before surgery; thus, this factor may have influenced the results. In addition, patients with relevant co-morbidities were excluded, and no differences in smoking habits were observed among the groups. Other factors, such as diet and periodontal status, should be addressed in future studies. Age is also a relevant factor influencing oral microbiome composition and may act as a confounder in our analysis. In our cohort, the observed age difference between healthy controls and patient groups is mainly attributable to the underlying clinical conditions, which are more prevalent in older individuals. We acknowledge that this imbalance represents a limitation of the study.

Furthermore, saliva may not fully reflect the microbial composition directly associated with necrotic bone lesions. Accordingly, results should be considered in the setting of the salivary microbiome as a surrogate of the oral environment, rather than a direct characterization of lesion-specific microbial communities.

Consequently, our results should be approached with caution, and further studies with more comprehensive control of these variables are warranted to better elucidate the relationship between osteonecrosis of the jaw and oral microbiome composition.

If confirmed in larger cohorts, it may be useful in the future to monitor the oral microbiome of patients receiving bisphosphonates or denosumab, to determine whether individuals with Streptococcus levels exceeding 5.51% are at higher risk of developing MRONJ. However, it should be noted that oral microbiome composition may itself be influenced by antibiotic administration.

## 5. Conclusions

In conclusion, this is the first preliminary study showing differences in terms of microbiota comparing healthy individuals, controls, and cases. Although these data are obtained in a limited cohort of patients, they could pave the way for further studies focused on the investigation of MRONJ predictors.

## Figures and Tables

**Table 1:** Characteristics of subjects enrolled in the study. Continuous variables were described as a median and interquartile range (IQR), whereas dichotomous variables as number and percentage.

	Controls		Cases		Healthy		*p*-Value (Between Cases and Controls)
No. of patients	21	38.2%	18	32.7%	16	29.1%	
Gender (male)	8	38.1	5	27.8	6	37.5%	0.496
Age, years	72	62; 78	76	69; 82.5	32	28; 53	0.622
BMI, kg/m^2^	25.8	21.9; 29.0	24.1	21.1; 28.8	23	21; 26	0.692
Leukocytes, ×10^9^ cells/L	5.62	4.99; 7.15	5.11	4.18; 7.59			0.286
Erythrocytes, ×10^6^ cells/μL	4.35	3.84; 4.50	4.20	3.40; 5.35			0.892
Hemoglobin, g/dL	12.35	10.48; 13.53	12.60	11.30; 13.20			0.725
Hematocrit, %	38.6	32.4; 41	38.3	35.8; 42.7			0.751
Platelets, ×10^9^ cells/L	234	159; 276	221	172.7; 323.7			0.817
Glycemia, mg/dL	95	76; 109	103	82.5; 132.5			0.427
Serum creatinine, mg/dL	0.92	0.63; 1.30	0.97	0.86; 1.14			0.696
Smoke	No: 14	66.7%	No: 12	66.7%	No: 14	87.5	0.526
	Yes: 3	14.3%	Yes: 3	16.7%	Yes: 2	12.5	
	In the past: 4	19.2%	In the past: 3	16.8%	In the past: 0		
Alcohol	8	38.1%	5	27.8%	1	6.3	0.428
Disease	osteoporosis 7	33.4%	osteoporosis 4	32.3%			0.293
	oncological 14	66.7%	oncological 14	77.7%			
Staging			1: 1	5.6%			
			2: 15	83.3%			
			3: 2	11.1%			

**Table 2:** Differences among the three groups in terms of presence of bacteria (dichotomous variable).

Type of Bacteria	*p*-Value	Healthy	Controls	Cases
*Veillonella montpellierensis*	0.013	+	+++	++
*Haemophilus parainfluenzae*	0.009	+++	+	++
*Veillonella parvula*	0.021	++	+	+++
*Streptococcus sanguinis*	0.005	+	+++	++
*Vibrio harveyi group*	0.024	+++	+	++
*Vibrionaceae*	0.002	+++	+	++
*Haemophilus*	0.004	++	+	+++
*Cobetia amphilecti*	0.018	+++	++	No
*Streptococcus mitis*	0.045	++	+++	+
*Bacteria*	<0.001	No	++	+++
*Neisseria perflava*	0.035	+++	++	No
*Porphyromonas pasteri*	0.043	++	+++	+
*Haemophilus* sp. *paraurethrae*	0.001	+++	++	+
*Fusobacterium*	0.005	+	++	+++
*Fusobacterium perodonticum*	0.005	+++	No	No
*Streptococcus gordoni*	0.028	+++	++	No
*Gemella haemolysans*	0.021	+++	No	No
*Fusobacterium nucleatum*	<0.001	+++	No	No
*Neisseria gonorrhoeae*	0.005	+++	No	No
*Unclassified rothia*	0.038	No	No	+++

**Table 3:** Differences among the three groups in terms of presence of bacteria (linear variable). +, ++ and +++ indicate the abundance of bacteria.

Type of Bacteria	*p*-Value	Healthy	Controls	Cases
*Veillonella parvula*	0.028	++	+	+++
*Haemophilus parainfluenzae*	0.034	+++	+	++

**Table 4:** Differences between controls and cases (linear variable) and receiving operated curve values. ++ and +++ indicate the abundance of bacteria.

Type of Bacteria	*p*-Value	ROC (Cut-Off Value; *p*-Value; Sensitivity; Specificity)	Controls	Cases
*Veillonella parvula*	0.042	2.55; <0.001; 75%; 75%	++	+++
*Streptococcus*	0.023	5.51; 0.009; 81%; 75%	++	+++
*Terrabacteria group*	0.043	4.1; <0.001; 75%; 75%	++	+++

**Table 5:** Regression analysis reporting factors predicting osteonecrosis of the jaw development.

Osteonecrosis of the Jaw Development				
	Univariate Regression		Multivariate Regression	
Predictive Factors	*p*-Value	OR (95% IC)	*p*-Value	OR (95% IC)
Gender	0.430	0.577 (0.147; 2.260)		
Age >50	0.647	1.789 (0.149; 21.541)		
*Terrabacteria* group >4.1%	0.178	9 (0.367; 221)		
*Streptococcus* >5.51%	0.007	13.5 (2.010; 90.689)	0.018	20 (01.655; 241.723)
*Veillonella parvula* >2.55%	0.053	21 (0.961; 458.842)		
Metronidazole Administration	0.049	9.5 (1.014; 88.966)		
Amoxicilline Administration	0.049	0.175 (0.031; 0.996)		
Body Mass Index	0.601	0.966 (0.848; 1.100)		

## Data Availability

The data presented in this study are available on request from the corresponding author. The data are not publicly available due to privacy.

## Abbreviations

The following abbreviations are used in this manuscript:

ONJ	Osteonecrosis of the Jaw
MRONJ	Medication-Related Osteonecrosis of the Jaw
BRONJ	Bisphosphonate-Related Osteonecrosis of the Jaw
AAOMS	American Association of Oral and Maxillofacial Surgeons
BMI	Body Mass Index
PCR	Polymerase Chain Reaction
16S rRNA	16S Ribosomal Ribonucleic Acid
ROC	Receiver Operating Characteristic
OR	Odds Ratio
CI	Confidence Interval
IQR	Interquartile Range
